# Crystallographic Evidence for Bi(I) as the Heaviest Halogen Bond Acceptor

**DOI:** 10.1021/jacs.4c11901

**Published:** 2024-10-18

**Authors:** Liam P. Griffin, Tim-Niclas Streit, Robin Sievers, Simon Aldridge, Rosa M. Gomila, Antonio Frontera, Moritz Malischewski

**Affiliations:** † Inorganic Chemistry Laboratory, Department of Chemistry, University of Oxford, South Parks Road, Oxford OX1 3QR, U.K.; ‡ Freie Universität Berlin, Institut für Anorganische Chemie, Fabeckstraße 34-36, D-14195 Berlin, Germany; § Department of Chemistry, Universitat de les Illes Balears, Crta de valldemossa km 7.5, 07122 Palma de Mallorca, Spain

## Abstract

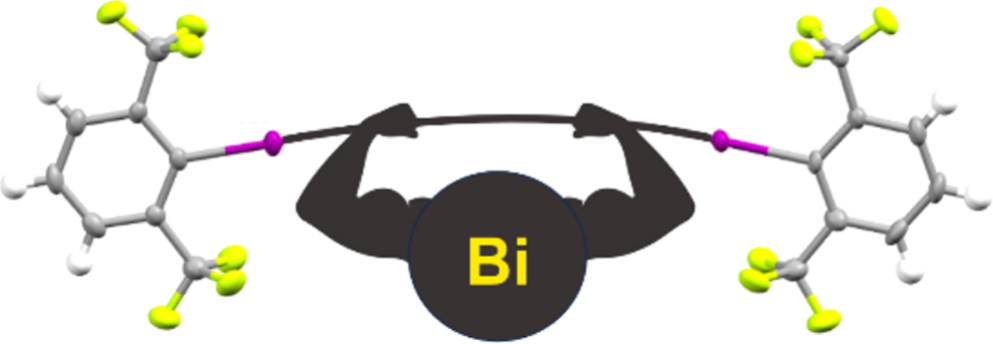

Complexation of the green bismuthinidene (RBi) with two equivalents of a highly fluorinated aryl iodide at low temperature allows the crystallographic identification of an unstable red species that can be regarded as an intermediate in an overall Bi(I) → Bi(III) oxidation process. Both C–I bonds are orientated toward the filled 6p orbital of bismuth (Bi–I distances 3.44–3.52 Å), leading to an elongation of the C–I bonds by 0.05 and 0.07 Å. Density functional theory (DFT) calculations confirm that the Bi(I) center is indeed acting as an electron donor, establishing two strong and directional halogen bonds. The color change from green to red upon halogen bond formation is a consequence of the energetic stabilization of a Bi(I) lone pair by interactions with the sigma-holes of the halogen bond donors. Overall, this study presents the first structural proof of bismuth, and more generally of heavy organopnictogen(I) compounds, acting as halogen bond acceptors.

## Introduction

In recent years, heavier main group elements have become increasingly popular for the activation of small molecules.^1^ In this context, bismuth is of great interest due to its low toxicity and rich redox chemistry, the latter showing parallels with transition metal chemistry.^2,3^ Recently, interest in the reduced oxidation state +I of bismuth has significantly increased. Pioneering work by Dostál has given access to bismuthinidenes, compounds in which the bismuth atom in the formal oxidation state +I is stabilized by a NCN pincer framework.^4,5^ The second lone pair associated with Bi(I) is localized in a p-orbital which lies perpendicular to the plane of the pincer ligand.^6^ Besides fundamental studies regarding the electronic structure of Bi(I) compounds^7,8^ the increased nucleophilicity of Bi(I) compounds allows them to act as donor ligands to metals.^9^ Furthermore, they have also been shown to mediate important organic transformations, e.g., transfer hydrogenations or hydro-defluorination reactions.^10,11^ Additionally, bismuthinidenes have been reported to undergo oxidative addition of alkyl iodides and aryl halides.^12,13^

Whereas halogen bonding between aryl iodides and nitrogen-based molecules has found widespread use in many areas of crystal engineering,^14−17^ compounds of the heavier pnictogens have rarely been used as halogen bond acceptors or electron donors overall.^18^ Only very few examples have been reported in the past years, e.g., using tertiary phosphines.^19−21^ In a seminal work, Friščić and Cinčić reported the successful cocrystallization of the halogen bond donor 1,3,5-trifluoro-2,4,6-triiodobenzene with Ph_3_P, Ph_3_As and Ph_3_Sb. However, no adduct could be isolated in the case of Ph_3_Bi.^22,23^ Recently, Bujak and Mitzel reported cocrystals of Me_3_As/Me_3_Sb with C_6_F_5_I.^24^ The fact that no solid-state structures with a bismuth compound acting as a halogen bond acceptor have been reported, and such structural motifs are typically not even considered in theoretical investigations,^25−27^ prompted us to pursue this synthetic challenge.

## Results and Discussion

**Figure 1:**
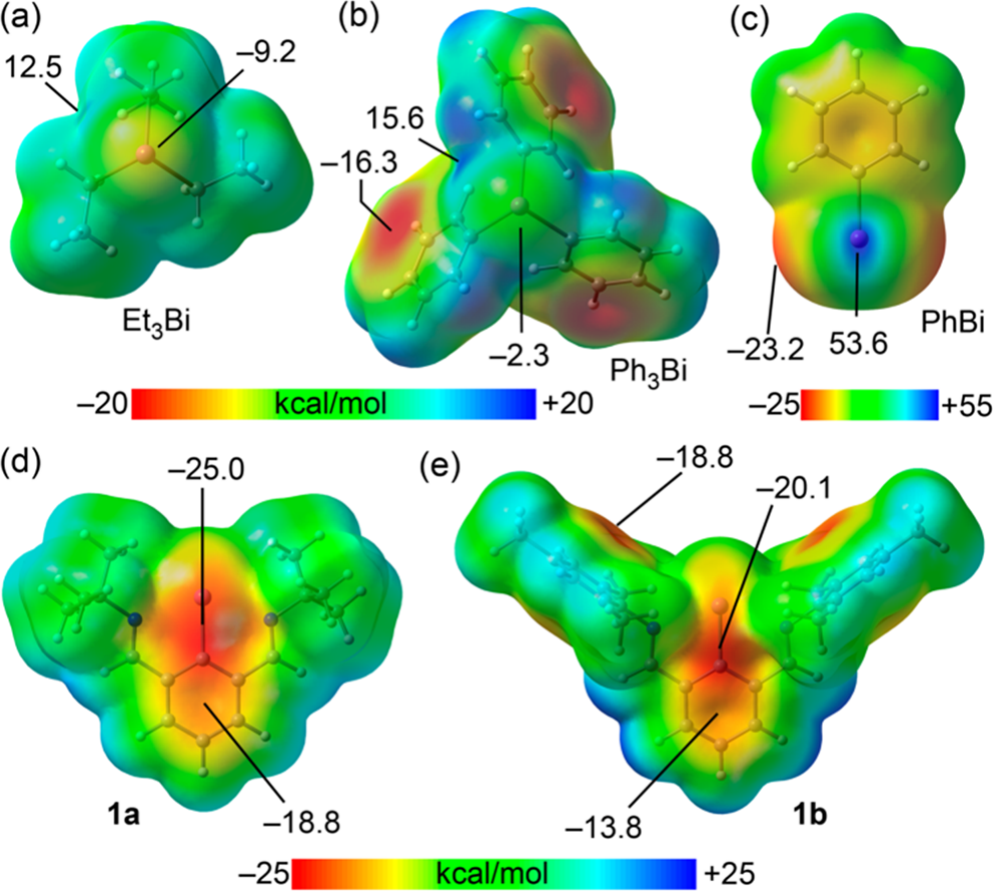
Molecular electrostatic potential (MEP) surfaces of Et_3_Bi (a), Ph_3_Bi (b), PhBi (c) and bismuthinidene compounds **1a** (d) and **1b** (e). Energies in kcal/mol.

The primary challenge with bismuth lies in the inert pair effect, which refers to the low energy of the 6s orbital, resulting in the diminished nucleophilicity of Bi(III) compounds. The Molecular Electrostatic Potential (MEP) surface plots of Et_3_Bi and Ph_3_Bi (Figure 1, top), reveal its unsuitability as a halogen bond acceptor. For Ph_3_Bi, the minima/electron-rich regions are concentrated on the aryl rings, with a MEP value of −16.3 kcal/mol, as compared to that of the bismuth, −2.3 kcal/mol. Despite the presence of electron-donating alkyl substituents in Et_3_Bi, its MEP value remains small (−9.2 kcal/mol). To counteract these modest MEP values, we shifted our focus to Bi(I) compounds instead of Bi(III). Nonetheless, the MEP of the basic PhBi model compound (in the singlet state) indicates an anisotropic MEP surface at Bi (Figure 1c). This surface displays two π-holes (positive areas) and two negative areas, attributed to the lone pair located in the p-type orbital aligned coplanar with the aromatic ring (see ESI for further discussion and Figure S4). As expected, the MEP values at these regions are significantly more negative (−23.2 kcal/mol) than those found in the Bi(III) compounds. However, the pronounced MEP maximum values (53.6 kcal/mol, representing π-holes) reveal Bi(I)’s dominant electrophilic nature over its nucleophilic one, rendering it unsuitable as a halogen bond acceptor. To circumvent this limitation, we considered using NCN-stabilized bismuthinidenes. We reasoned that the LPs on the imine N atoms could engage with the π-holes at Bi, positioning the stereoactive lone pairs above and below the plane of the aromatic ring (as shown in Figure 1d,e). Within this configuration, Bi(I) demonstrates pronounced nucleophilicity, as evidenced by a MEP value of −25.0 kcal/mol in the case of the *tert*-butyl-derivative **1a** or −20.1 kcal/mol for the mesityl derivative **1b** (although in this case the nucleophilic regions are less accessible and displaced toward the aromatic ring).

As halogen bond donors, a variety of fluorinated organo-iodides were deemed suitable due to their pronounced σ-holes.^18,28−30^ The main synthetic challenge of our approach to combine Bi(I) compounds with fluorinated organoiodides was to find a suitable combination in which the bismuth compound would be basic enough to form a halogen-bonded adduct with Bi···I interactions, while not being so electron-rich as to facilitate oxidation to Bi(III) or cleavage of the C–I bond to form a I–Bi–C moiety. In this context, the use of fluorinated alkyl iodides seemed less promising due to their higher inherent reactivity due to weaker C–I bonds.

**Figure 2:**
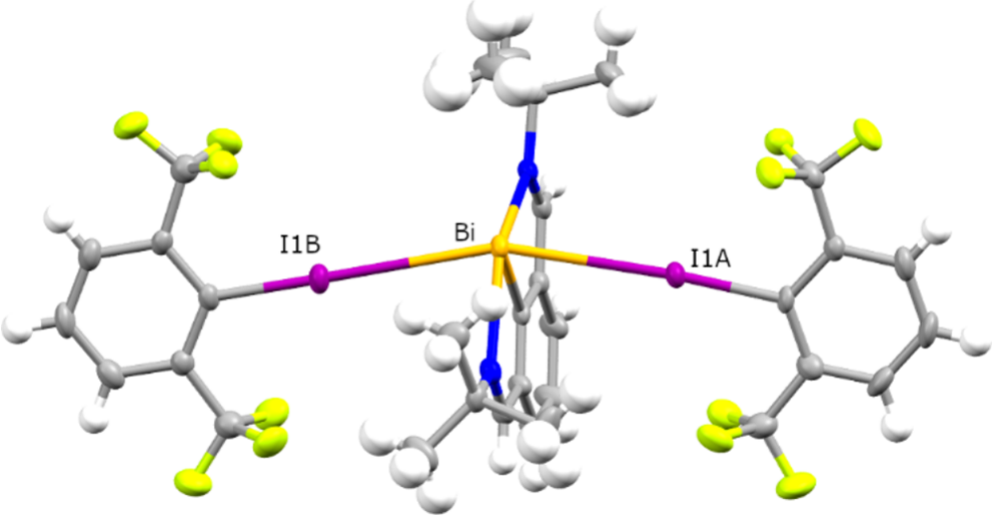
Molecular structure of **3**, ellipsoids shown at 50% probability, color code: hydrogen white, carbon gray, fluorine yellow-green, nitrogen blue, bismuth dark yellow, iodine purple.

**Scheme 1:**
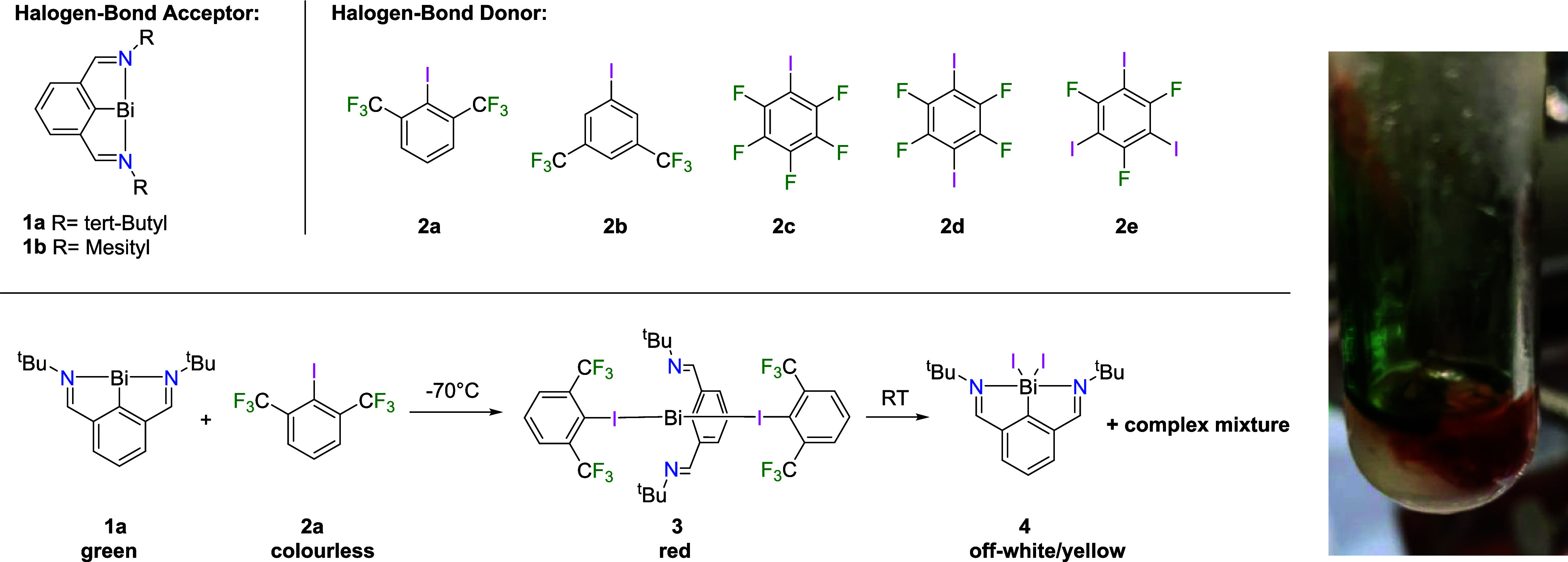
List of Utilized Precursors and Formation of the Crystalline Halogen-Bonded Adduct **3** and a Photo of a Typical Color Change from Green to Red Upon Mixing Aryl Iodides with **1a**

By adding precooled suspensions of fluorinated aryl iodides in hexane to solutions of the green bismuthinidenes **1a** or **1b** at −70 °C we targeted the respective halogen-bonded adducts (Scheme 1). Whereas no color change was observed for the mesityl derivative **1b** with any aryl iodide, color changes to orange-red were observed for the *tert*-butyl-substituted compound **1a** within minutes for all except **2b** – the only aryl iodide without ortho-substituents. Interestingly, this red color was never observed when solvents other than alkanes were used. In general, warming of the aryl iodide/bismuthinidene mixtures to temperatures in the range of −60 to −40 °C led to disappearance of the green/red color, and yellow reaction mixtures where obtained which we attribute to decomposition/oxidation to Bi(III). Typically, these intermediately formed red species showed very low solubility in hexane, and only in case of 2,6-bis(trifluoromethyl)iodobenzene **2a** was a significant red coloration of the hexane solution visible in the cold. By carefully increasing the reaction temperature to −65 °C, followed by slow cooling in a −78 °C freezer, red crystals of the targeted adduct **3** were obtained.^31^ Warming to room temperature, by contrast, gave a complex product mixture (Figure S1) which contained small amounts of literature-known RBiI_2_**4** (identified by XRD).^12^ The highly unstable red species **3** crystallizes in the monoclinic space group *P*2_1_/*n*, and the solid-state structure obtained by X-ray crystallography reveals a trinuclear complex formed by interaction of two intact aryl iodide molecules with the bismuth center. The alignment of the bismuth center and the two iodine atoms is almost linear (I–Bi–I angle of 160.659(12)°), implying the presence of interactions with the filled p-orbital of bismuth perpendicular to the NCN plane (Figure 2).

The distances between bismuth and iodine are 3.4382(5) Å (I1A) and 3.5226(5) (I1B), i.e., significantly below the sum of the respective van der Waals radii (4.05 Å),^32^ and correspond to RXB values of 0.849 and 0.870.^33^ Interestingly, the pnictogen-iodine distances are similar to or even shorter than in the adducts Ph_3_As·C_6_I_3_F_3_ (3.4211(3) Å), Ph_3_Sb·C_6_I_3_F_3_ (3.5747(3) Å)^17^ or Me_3_Sb·C_6_F_5_I (3.4951(4) Å).^18^ With regards to the C–I bond lengths (C1A-I1A 2.171(3) Å and C1B-I1B 2.147(4) Å), a significant increase is observed when compared with free C_6_H_3_(CF_3_)_2_I (2.100(5) A), consistent with population of the σ*(C–I) orbital.^34^

In addition to the electron-withdrawing effect of the CF_3_ groups, we hypothesized that their placement in the *ortho*-positions would additionally provide the possibility for weak H···F contacts with the *tert*-butyl groups of the bismuthinidene to augment the halogen bonded assembly. However, the crystal structure shows that these moieties are, in the main, too distant from each other: only one such contact (F2B-H14A 2.607(3) Å) is observed. Instead, intermolecular H···F contacts between the CF_3_ groups and the hydrogen atoms of the *tert*-butyl groups (F2A-H15B 2.587(2) Å) and the aryl ring of the bismuthinidene are observed (H3-F5B 2.564(3) Å, H5-F2A 2.465(3) Å) (see Figure S2). Furthermore, the four CF_3_ groups in the assembly form strong intramolecular hydrogen bonds to the hydrogen substituents in the ortho-position (2.277(2)–2.283(3) Å).

We have examined the potential halogen bonds present in the bismuthinidene·2,6-bis(trifluoromethyl)iodobenzene adduct **3** using density functional theory (DFT) calculations. Initially, we compared the geometry of the halogen-bonded (HaB) adduct in the solid state with its optimized counterparts (Figure S3, SI). Specifically, two DFT geometry optimizations were undertaken: one for the isolated adduct in the gas phase and another employing periodic boundary conditions (PBC) to account for packing effects. Notably, the gas phase geometry closely resembles both the experimental and PBC geometries, with the latter two being nearly identical regarding the relative orientation of the crystal conformers (Figure S3). Importantly, the I–Bi halogen bonds persist in the gas phase with closely analogous distances (3.456 and 3.459 Å). This observation underscores the structure-directing capability of the halogen bonds and refutes any notion that they could be manifested merely due to packing effects. The primary variance between the gas phase and the experimental/PBC configurations is evident in the I–Bi–I angle. In the gas phase, this angle is calculated to be 147.9°, compared to 161.6° for PBC and 160.6° for the X-ray study. This reduced angle in the gas phase arises as the isolated adduct attempts to maximize secondary intramolecular interactions.

**Figure 3:**
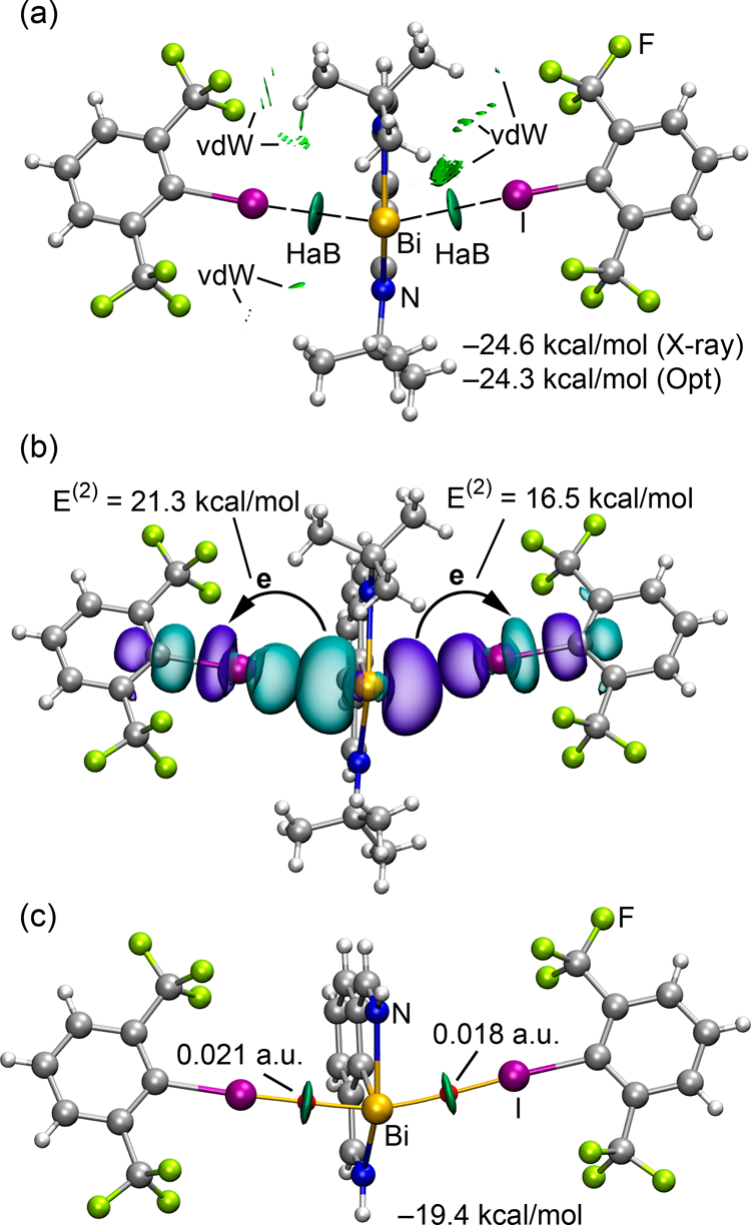
(a) NCIplot analysis of compound **3**. Only intermolecular interactions are represented. (b) NBOs involved in the LP(Bi)→ σ*(C–I) charge transfer. The second order perturbation energies E^(2)^ are indicated. (c) Combined QTAIM (BCP as red spheres) and NCIplot analysis of the mutated compound (*t*Bu → H). The density values at the bond critical points are indicated.

Figure 3a presents the noncovalent interaction plot (NCIplot) of compound **3**. The reduced density gradient (RDG) iso-surfaces provide a visual representation of interactions in real space. Dual disk-shaped RDG iso-surfaces are observed between the Bi and I atoms, corroborating the presence of halogen bonds. Additionally, the NCIplot highlights (in green) RDG iso-surfaces between the methyl-trifluoromethyl groups and between the methyl-iodine atoms, signifying weak van der Waals (vdW) interactions (Figure 3). When examining the formation energy of the adduct against isolated monomers, values of −24.6 kcal/mol (experimental geometry) and −24.3 kcal/mol (DFT-optimized, isolated adduct) have been calculated. This finding further affirms the assertion that packing effects are not the primary force behind adduct formation.

Natural bond orbital (NBO) analysis has been used to probe the significance of orbital donor-acceptor interactions within the halogen bonds. This analysis reveals that the two bismuth LPs reside in the 6s and 6p atomic orbitals. The LP within the 6p orbital participates in electron donation from bismuth to the antibonding σ*(C–I) orbitals (Figure 3b). The LP(Bi) → σ*(C–I) charge transfer energies (21.3 and 16.5 kcal/mol), further reinforce the idea of predominant system stabilization arising from the HaB formation. To evaluate the HaB energies independent of the influence of vdW interactions, we also modeled a mutated adduct, substituting *tert*-butyl groups with H atoms. This effectively eliminates the CF_3_···H_3_C and CH_3_···I interactions. Figure 3c depicts this model, integrating both the quantum theory of atoms-in-molecules (QTAIM) and NCIPlot analyses. These methods confirm the exclusive establishment of HaBs in the mutated adduct, each characterized by a bond critical point (BCP) and bond path linking the I and Bi atoms. The electron density values at the BCPs are consistent with strong halogen bonds.^35^ The interaction energy diminishes to −19.4 kcal/mol, relative to a value of −24.6 kcal/mol for the “full” system. Such findings emphasize the assertion that the formation energy is predominantly attributed to the I···Bi interactions, aligning with the pronounced and negative MEP value observed at the Bi atom, as visualized in Figure 1d.

**Figure 4:**
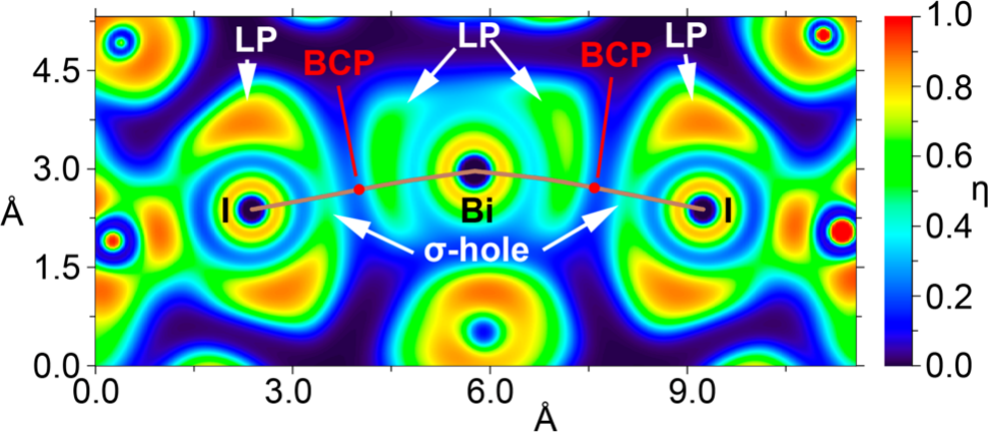
Electron localization function of compound **3** represented in the I–Bi–I plane. The bond paths are represented as brown lines and the BCPs as red dots.

The two-dimensional (2D) electron localization function (ELF) plot of compound **3** is depicted in Figure 4, offering further insight into the role(s) of the σ-holes in the interactions. This figure provides a sectional view of the ELF 2D map, focusing on the plane demarcated by the Bi atom and its two interacting iodine counterparts. Through this ELF visualization, it becomes evident that the σ-holes on the iodine atoms are oriented toward the LP of the bismuth atom. Indeed, the bond path connecting I to Bi passes through both the iodine σ-hole and bismuth LP. This specific electron localization in the I–Bi–I plane at the Bi atom aligns well with the findings from the NBO analysis, particularly emphasizing the involvement of the LP located at the atomic 6p orbital.

The potential involvement of the C_ipso_ in halogen bonding in compound **3** has been ruled out based on the absence of RDG isosurfaces, BCPs, and bond paths connecting the C_ipso_ and I atoms, as well as ELF analysis, which shows the iodine σ-holes directed toward the lone pairs on Bi. The only indication is a minimal σ(Bi–C) → σ*(C–I) charge transfer in the NBO analysis (0.3 kcal/mol, see Figure S5, SI), which is negligible compared to the LP(Bi) → σ*(C–I) charge transfer (see Figure 3b).

**Figure 5:**
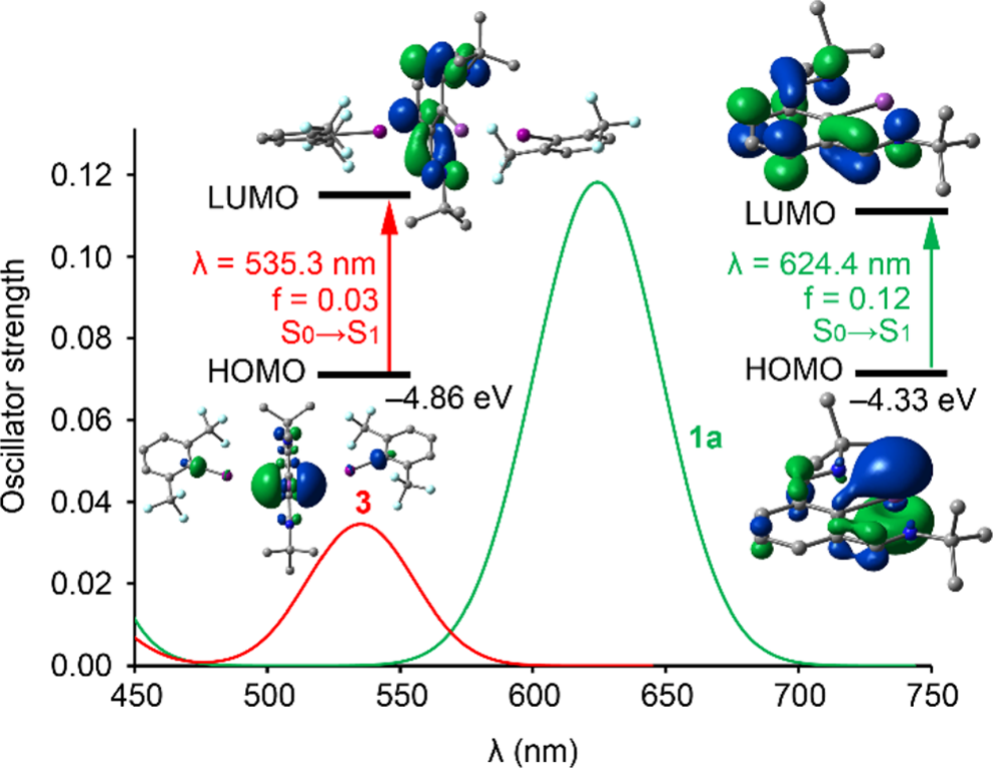
Theoretical absorption spectra in the visible region of compound **1a** (green line) and compound **3** (red line). The transition orbitals, wavelengths (λ), and oscillator strengths are indicated. The energies of the HOMOs are also provided.

To understand the origin of the red color of the highly unstable adduct **3** and determine if it is related to the formation of the Bi–I interactions, the ultraviolet–visible (UV–vis) spectrum of **3** was calculated (see SI for details). As shown in Figure 5, the theoretical spectra in the visible region align well with experimental results, predicting a green color for bismuthinidene **1a** and a red color for adduct **3**. In both cases, the lowest-lying transition corresponds to an S_0_ → S_1_ excitation (highest occupied molecular orbital (HOMO) → lowest unoccupied molecular orbital (LUMO)) involving an LP → π* charge transfer. The formation of Bi–I interactions lowers the HOMO energy (LP at Bi) in **3**, increasing the HOMO–LUMO gap with respect to **1a** and causing the color change from green to red, consistent with experimental findings.

## Conclusions

In summary, we provide the first crystallographic and quantum chemical evidence for Bi(I) as the heaviest halogen bond acceptor. The crystal structure of this highly unstable adduct **3** displays Bi···I distances significantly below the sum of van der Waals radii as well as elongated C–I bonds within the fluorinated aryl iodide moieties. Consequently, this crystal structure can be regarded as a snapshot of an intermediate formed during a reaction between an aryl iodide and a low valent main group element just before the C–I bond breaking occurs. In line with the low stabilities of such adducts, the structural characterization of other halogen-bonded adducts between the *tert*-butyl bismuthinidene **1a** and other fluorinated aryl iodides was not possible due to the restrictions enforced by using only alkane solvents (at low temperatures), and the resulting low solubilities of the compounds. Nevertheless, the intense red color of all these combinations suggests adduct formation, in line with the calculated UV–vis spectrum of **3**. Why no color change was observed during cocrystallization experiments of the mesityl compound **1b** and fluorinated aryl iodides cannot be explained beyond doubt without a crystal structure. It is possible that the interactions between the electron-rich aromatic mesityl rings and the electron-poor fluorinated aryl iodides enforce a different intermolecular arrangement, which counteracts the Bi–I interaction. This is supported by the MEP surface of compound **1b** (see Figure 1e), which shows a less accessible and less nucleophilic Bi atom, along with two accessible negative regions over the mesityl π-systems. However, it could also be that no cocrystallization is possible at all due to the weaker intermolecular interactions.

Nonetheless, this study not only sheds light on the unique potential of bismuth compounds in halogen bonding but also paves the way for further exploration of the realm of heavy main group elements, yielding an isolated reactive intermediate of central importance to Bi(I) centered catalysis. Our findings underscore the utility of comprehensive computational and experimental investigations to truly understand the potential of these unique elements in molecular chemistry. Furthermore, the results emphasize the importance of considering alternative oxidation states and molecular frameworks to unlock unexpected bonding and reactivity avenues, as is evident from the successful employment of the Bi(I) state in halogen bonding. As the field continues to grow and diversify, we anticipate that the lessons from this study will serve as a foundational reference, inspiring chemists to explore the uncharted territories of main group element chemistry.

## Supporting Information Available

Experimental and computational details and crystallographic data (PDF)

## References

[ref1] Oberdorf K.; Lichtenberg C. Small molecule activation by well-defined compounds of heavy p-block elements. Chem. Commun. 2023, 59 (52), 8043–8058. 10.1039/D3CC02190D. 37284835

[ref2] Moon H. W.; Cornella J. Bismuth Redox Catalysis: An Emerging Main-Group Platform for Organic Synthesis. ACS Catal. 2022, 12 (2), 1382–1393. 10.1021/acscatal.1c04897. 35096470 PMC8787757

[ref3] Mato M.; Spinnato D.; Leutzsch M.; Moon H. W.; Reijerse E. J.; Cornella J. Bismuth radical catalysis in the activation and coupling of redox-active electrophiles. Nat. Chem. 2023, 15 (8), 1138–1145. 10.1038/s41557-023-01229-7. 37264103 PMC10396954

[ref4] Dostál L. Quest for stable or masked pnictinidenes: Emerging and exciting class of group 15 compounds. Coord. Chem. Rev. 2017, 353, 142–158. 10.1016/j.ccr.2017.10.009.

[ref5] Šimon P.; de Proft F.; Jambor R.; Růzicka A.; Dostál L. Monomeric organoantimony(I) and organobismuth(I) compounds stabilized by an NCN chelating ligand: syntheses and structures. Angew. Chem., Int. Ed. 2010, 49 (32), 5468–5471. 10.1002/anie.201002209. 20602393

[ref6] Vránová I.; Alonso M.; Lo R.; Sedlák R.; Jambor R.; Růžička A.; De Proft F.; Hobza P.; Dostál L. From Dibismuthenes to Three- and Two-Coordinated Bismuthinidenes by Fine Ligand Tuning: Evidence for Aromatic BiC3N Rings through a Combined Experimental and Theoretical Study. Chem. - Eur. J. 2015, 21 (47), 16917–16928. 10.1002/chem.201502724. 26434943

[ref7] Mukhopadhyay D. P.; Schleier D.; Wirsing S.; Ramler J.; Kaiser D.; Reusch E.; Hemberger P.; Preitschopf T.; Krummenacher I.; Engels B.; Fischer I.; Lichtenberg C. Methylbismuth: an organometallic bismuthinidene biradical. Chem. Sci. 2020, 11 (29), 7562–7568. 10.1039/D0SC02410D. 32874526 PMC7450715

[ref8] Pang Y.; Nöthling N.; Leutzsch M.; Kang L.; Bill E.; van Gastel M.; Reijerse E.; Goddard R.; Wagner L.; SantaLucia D.; DeBeer S.; Neese F.; Cornella J. Synthesis and isolation of a triplet bismuthinidene with a quenched magnetic response. Science 2023, 380 (6649), 1043–1048. 10.1126/science.adg2833. 37200451

[ref9] Vránová I.; Alonso M.; Jambor R.; Růžička A.; Erben M.; Dostál L. Stibinidene and Bismuthinidene as Two-Electron Donors for Transition Metals (Co and Mn). Chem. - Eur. J. 2016, 22 (22), 7376–7380. 10.1002/chem.201601272. 26994732

[ref10] Pang Y.; Leutzsch M.; Nöthling N.; Katzenburg F.; Cornella J. Catalytic Hydrodefluorination via Oxidative Addition, Ligand Metathesis, and Reductive Elimination at Bi(I)/Bi(III) Centers. J. Am. Chem. Soc. 2021, 143 (32), 12487–12493. 10.1021/jacs.1c06735. 34358426 PMC8377712

[ref11] Wang F.; Planas O.; Cornella J. Bi(I)-Catalyzed Transfer-Hydrogenation with Ammonia-Borane. J. Am. Chem. Soc. 2019, 141 (10), 4235–4240. 10.1021/jacs.9b00594. 30816708 PMC6728098

[ref12] Mato M.; Bruzzese P. C.; Takahashi F.; Leutzsch M.; Reijerse E. J.; Schnegg A.; Cornella J. Oxidative Addition of Aryl Electrophiles into a Red-Light-Active Bismuthinidene. J. Am. Chem. Soc. 2023, 145 (34), 18742–18747. 10.1021/jacs.3c06651. 37603853 PMC10472430

[ref13] Hejda M.; Jirásko R.; Růžička A.; Jambor R.; Dostál L. Probing the Limits of Oxidative Addition of C(sp 3)–X Bonds toward Selected N,C,N -Chelated Bismuth(I) Compounds. Organometallics 2020, 39 (23), 4320–4328. 10.1021/acs.organomet.0c00418.

[ref14] Metrangolo P.; Neukirch H.; Pilati T.; Resnati G. Halogen bonding based recognition processes: a world parallel to hydrogen bonding. Acc. Chem. Res. 2005, 38 (5), 386–395. 10.1021/ar0400995. 15895976

[ref15] Metrangolo P.; Resnati G. Halogen Bonding: A Paradigm in Supramolecular Chemistry. Chem. - Eur. J. 2001, 7 (12), 2511–2519. 10.1002/1521-3765(20010618)7:123.0.co;2-t. 11465442

[ref16] Metrangolo P.; Meyer F.; Pilati T.; Resnati G.; Terraneo G. Halogen bonding in supramolecular chemistry. Angew. Chem., Int. Ed. 2008, 47 (33), 6114–6127. 10.1002/anie.200800128. 18651626

[ref17] Gilday L. C.; Robinson S. W.; Barendt T. A.; Langton M. J.; Mullaney B. R.; Beer P. D. Halogen Bonding in Supramolecular Chemistry. Chem. Rev. 2015, 115 (15), 7118–7195. 10.1021/cr500674c. 26165273

[ref18] Liyanage S.; Ovens J. S.; Scheiner S.; Bryce D. L. Tuneable tetrel bonds between tin and heavy pnictogens. Chem. Commun. 2023, 59 (58), 9001–9004. 10.1039/D3CC02644B. 37401672

[ref19] Zheng D. N.; Szell P. M. J.; Khiri S.; Ovens J. S.; Bryce D. L. Solid-state multinuclear magnetic resonance and X-ray crystallographic investigation of the phosphorus···iodine halogen bond in a bis(dicyclohexylphenylphosphine)(1,6-diiodoperfluorohexane) cocrystal. Acta Crystallogr., Sect. B: Struct. Sci., Cryst. Eng. Mater. 2022, 78 (3-2), 557–563. 10.1107/S2052520622004322. 35702972

[ref20] Siegfried A. M.; Arman H. D.; Kobra K.; Liu K.; Peloquin A. J.; McMillen C. D.; Hanks T.; Pennington W. T. Phosphorus···Iodine Halogen Bonding in Cocrystals of Bis(diphenylphosphino)ethane (dppe) and p -Diiodotetrafluorobenzene (p -F 4 DIB). Cryst. Growth Des. 2020, 20 (11), 7460–7469. 10.1021/acs.cgd.0c01129.

[ref21] Xu Y.; Huang J.; Gabidullin B.; Bryce D. L. A rare example of a phosphine as a halogen bond acceptor. Chem. Commun. 2018, 54 (78), 11041–11043. 10.1039/C8CC06019C. 30215643

[ref22] Linden A.; Luan X.; Wolstenholme D.; Dorta R. CSD Communications, CCDC 1999976. 2020.

[ref23] Lisac K.; Topić F.; Arhangelskis M.; Cepić S.; Julien P. A.; Nickels C. W.; Morris A. J.; Friščić T.; Cinčić D. Halogen-bonded cocrystallization with phosphorus, arsenic and antimony acceptors. Nat. Commun. 2019, 10 (1), 61 10.1038/s41467-018-07957-6. 30610194 PMC6320372

[ref24] Bujak M.; Stammler H.-G.; Vishnevskiy Y. V.; Mitzel N. W. Very close I···As and I···Sb interactions in trimethylpnictogen-pentafluoroiodobenzene cocrystals. CrystEngComm 2021, 24 (1), 70–76. 10.1039/D1CE01268A.

[ref25] Ping N.; Zhang H.; Meng L.; Zeng Y. Insight into the halogen-bonding interactions in the C6F5X···ZH3 (X = Cl, Br, I; Z = N, P, As) and C6F5I···Z (Ph)3 (Z = N, P, As) complexes. Struct. Chem. 2021, 32 (2), 767–774. 10.1007/s11224-020-01656-z.

[ref26] Amonov A.; Scheiner S. Heavy pnicogen atoms as electron donors in sigma-hole bonds. Phys. Chem. Chem. Phys. 2023, 25 (35), 23530–23537. 10.1039/D3CP03479H. 37656119

[ref27] Hong Y.; Lu Y.; Zhu Z.; Xu Z.; Liu H. Metalloids as halogen bond acceptors: A combined crystallographic data and theoretical investigation. Chem. Phys. Lett. 2020, 745, 137270 10.1016/j.cplett.2020.137270.

[ref28] Rozhkov A. V.; Novikov A. S.; Ivanov D. M.; Bolotin D. S.; Bokach N. A.; Kukushkin V. Y. Structure-Directing Weak Interactions with 1,4-Diiodotetrafluorobenzene Convert One-Dimensional Arrays of [M II (acac) 2] Species into Three-Dimensional Networks. Cryst. Growth Des. 2018, 18 (6), 3626–3636. 10.1021/acs.cgd.8b00408.

[ref29] Johnson M. T.; Džolić Z.; Cetina M.; Wendt O. F.; Ohrström L.; Rissanen K. Neutral Organometallic Halogen Bond Acceptors: Halogen Bonding in Complexes of PCPPdX (X = Cl, Br, I) with Iodine (I(2)), 1,4-Diiodotetrafluorobenzene (F4DIBz), and 1,4-Diiodooctafluorobutane (F8DIBu). Cryst. Growth Des. 2012, 12 (1), 362–368. 10.1021/cg201170w. PMC3252960 22229019

[ref30] Aakeröy C. B.; Baldrighi M.; Desper J.; Metrangolo P.; Resnati G. Supramolecular hierarchy among halogen-bond donors. Chem. - Eur. J. 2013, 19 (48), 16240–16247. 10.1002/chem.201302162. 24130038

[ref31] Deposition Numbers 23086002308601 contain the supplementary crystallographic data for this paper. These data are provided free of charge by the joint Cambridge Crystallographic Data Centre and Fachinformationszentrum Karlsruhe Access Structures service.

[ref32] Mantina M.; Chamberlin A. C.; Valero R.; Cramer C. J.; Truhlar D. G. Consistent van der Waals radii for the whole main group. J. Phys. Chem. A 2009, 113 (19), 5806–5812. 10.1021/jp8111556. 19382751 PMC3658832

[ref33] RXB = d(X···Y)/(rX + rY); d(X···Y) is the distance between X and Y in an R–X···Y halogen bond; rX and rY are the respective vdW radii of X and Y.

[ref34] Linden A.; Luan X.; Wolstenholme D.; Dorta R. CSD Communications, CCDC 1999976, 2020.

[ref35] Bartashevich E. V.; Tsirelson V. G. Interplay between non-covalent interactions in complexes and crystals with halogen bonds. Russ. Chem. Rev. 2014, 83 (12), 1181–1203. 10.1070/RCR4440.

