# Supplementary material for: Crystallographic Evidence for Bi(I) as the Heaviest Halogen Bond Acceptor
Source: J Am Chem Soc. 2024 Oct 18;146(43):29877–82. doi: 10.1021/jacs.4c11901 (PMC11528405; doi:10.1021/jacs.4c11901)
Supplement: Supplementary file 1 — ja4c11901_si_001.pdf [file ja4c11901_si_001.pdf]

Crystallographic evidence for Bi(I) as the heaviest halogen bond acceptor

Liam P. Griffin[§], Tim-Niclas Streit[§], Robin Sievers, Simon Aldridge, Rosa M. Gomila, Antonio Frontera*, Moritz Malischewski*

[§] Liam P. Griffin and Tim-Niclas Streit contributed equally to this manuscript.

Table of Contents

Synthetic procedures.. 2
X-ray diffraction (XRD) 3
Computational Details 7
Cartesian Coordinates... 8
References... 15

Synthetic procedures

Manipulations were carried out using standard Schlenk line and glove box techniques under atmosphere of argon. Solvents were degassed and stored over Na_2K.

Bismuthinidene **1a[1] and 1b[1]** and 2,6-trifluoromethyl-iodobenzene **2**[2] were synthesized according to literature procedures. Other fluorinated aryl iodides were obtained from chemical suppliers.

General procedure

-70°C slurries of green bismuthinidenes **1a** or **1b** in hexane were mixed with similarly cold solutions/slurries of fluorinated aryl iodides **2a-2e** in hexane. The green colour of **1b** persists in presence of the fluorinated aryl iodides in the cold (-60°C) but no red colour is observed. Upon warming the green colour disappears and the mixture turns yellow. In case of **1a**, at temperatures below -60°C, appearance of a red colour is observed within minutes when combined with aryl iodides **2a**, **2c**, **2d**, **2e**. In case of **2b** the mixture stays green. However, the red products rapidly precipitate from the cold solutions. Only in case of **2a** (see below) some solubility of the red product is observed. Upon warming above -60°C the red colour of the mixtures rapidly disappears and the mixtures turn yellow (unspecified decomposition).

Isolation of halogen-bonded intermediate **3**

-70 ºC hexane slurries of both 2,6-Bis(trifluoromethyl)iodobenzene **2a** (23 mg (68 μmol = 3 eq) in 1.5 ml) and green bismuthinidene **1** (10 mg (22 μmol = 1 eq) in 1.5 ml) were prepared. The slurry of **1** was gently warmed and stirred until just dissolved, before rapid addition to the slurry of 2,6-bis(trifluoromethyl)iodobenzene. After a few minutes, a red microcrystalline precipitate could be observed. The reaction mixture was warmed to -65 ºC at which point the red precipitate had fully redissolved. Slow cooling and storage overnight at -78 ºC yielded red crystalline blocks of **3** suitable for single crystal X-ray diffraction analysis.

Warming of a sample of **3** to room temperature

An *in situ* generated sample of **3** (prepared as described above) was slowly warmed to room temperature, at which point the red colour slowly faded to a bright yellow. ^{1}H NMR indicated that this thermal decomposition was highly unselective, yielding a complex mixture of species (see Figure S1 below).

Carrying out the above procedure instead in toluene (3 ml) leads to no formation of any red colour in either the solid or solution phase. Warming results in conversion of the green initial solution directly to a bright yellow solution. Storage of the resulting yellow solution at room temperature for several days yielded crystalline yellow rods

which were crystallographically shown to be the literature-known Bi(III) species $RBiI_2$.[3]

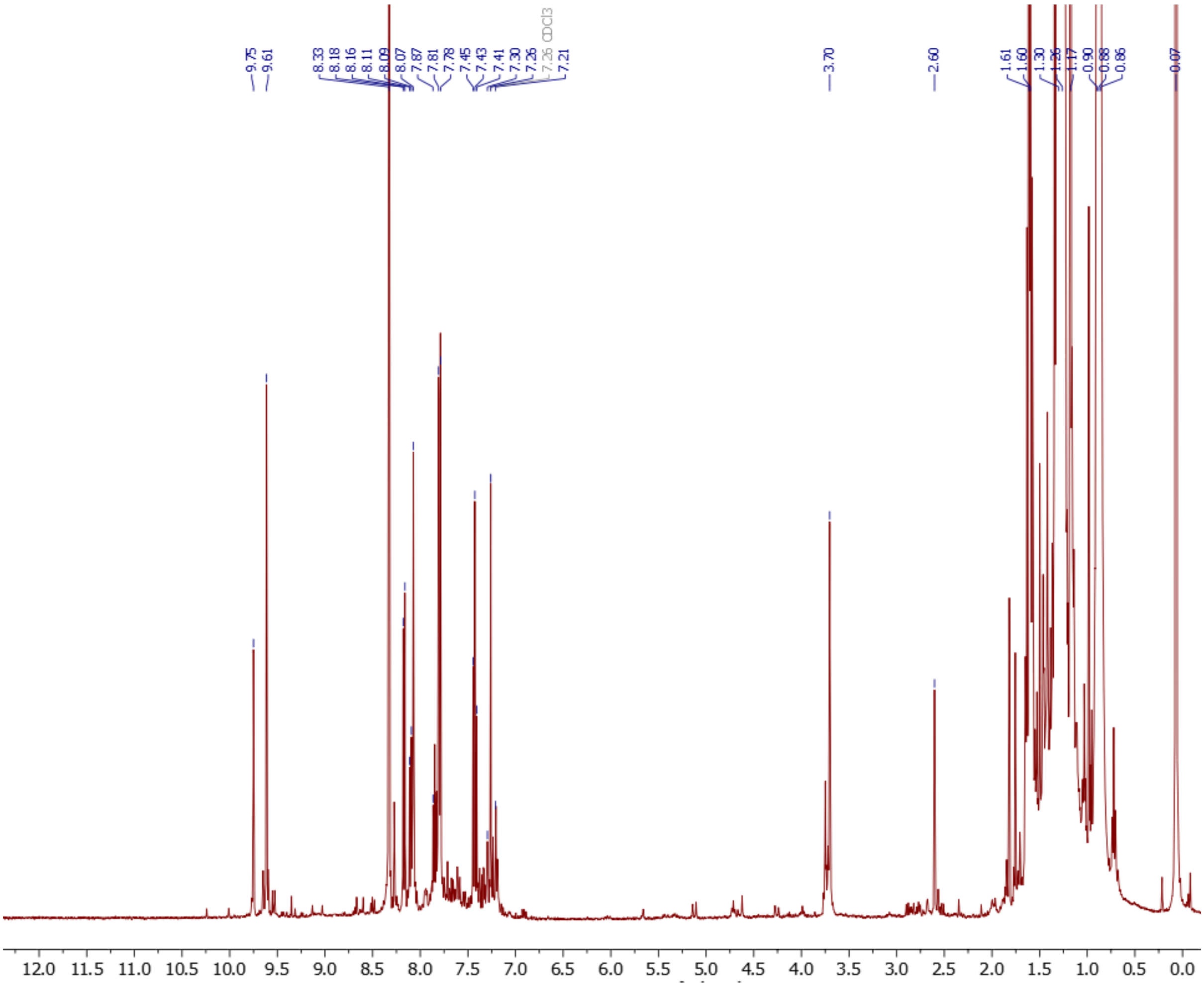

Figure S1: Crude NMR in $CDCl_3$ of the product mixture of **1a** and **2a** after warming to room temperature

X-ray diffraction (XRD)

X-Ray data were collected on a BRUKER D8 Venture system. Data were collected at 105(2) K using graphite monochromated Mo K_α radiation (λ_α = 0.71073 Å). The strategy for the data collection was evaluated by using the Smart software. The data were collected by the standard “ψ-ω scan techniques” and were scaled and reduced using Saint+software. The structures were solved by using Olex2,[4] the structure was solved with the XT[5] structure solution program using Intrinsic Phasing and refined with the XL refinement package[6] using Least Squares minimization. Bond length and angles were measured with Diamond Crystal and Molecular Structure Visualization Version 4.6.2.[7] Drawings were generated with Mercury[8] and POV-Ray.[9]

Table 1. Crystallographic data of **3**.

Identification code	2308600
Empirical formula	$C_{32}H_{29}BiF_{12}I_2N_2$
Formula weight	1132.35
Temperature/K	105.00
Crystal system	monoclinic
Space group	$P2_1/n$
a/Å	13.4138(13)
b/Å	9.2197(8)
c/Å	29.596(3)
α/°	90
β/°	98.623(4)
γ/°	90
Volume/Å^3	3618.8(6)
Z	4
ρ_{calc}g/cm^3	2.078
μ/mm^{-1}	6.666
F(000)	2128.0
Crystal size/mm^3	0.1 × 0.1 × 0.1
Radiation	MoKα (λ = 0.71073)
2Θ range for data collection/°	4.632 to 56.564
Index ranges	-17 ≤ h ≤ 17, -12 ≤ k ≤ 10, -38 ≤ l ≤ 39
Reflections collected	29869
Independent reflections	8939 [R_{int} = 0.0352, R_{sigma} = 0.0409]
Data/restraints/parameters	8939/0/448
Goodness-of-fit on F^2	1.057
Final R indexes [I>=2σ (I)]	R_1 = 0.0254, wR_2 = 0.0552
Final R indexes [all data]	R_1 = 0.0367, wR_2 = 0.0636
Largest diff. peak/hole / e Å^{-3}	0.69/-1.36

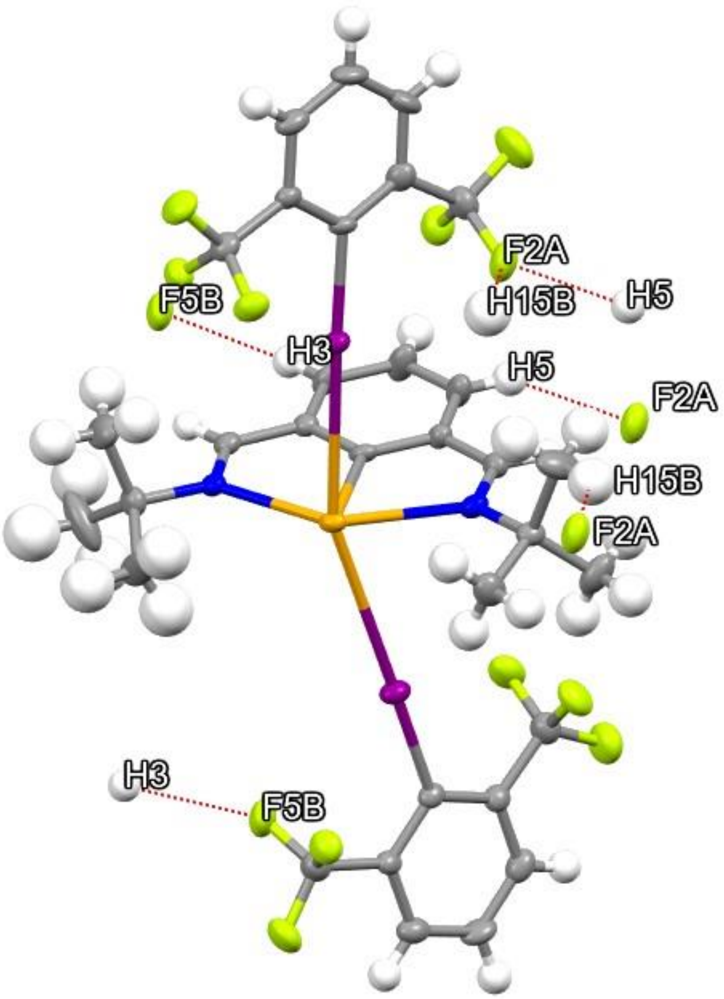

Figure S2. Intermolecular H···F contacts (indicated with red dots) in the crystal structure of **3**, ellipsoids drawn at 50% probability, colour code: hydrogen white, carbon grey, fluorine yellow-green, nitrogen blue, bismuth dark yellow, iodine purple.

Table 2. Crystallographic data of **4**.(was already reported as CCDC 2259026)[3b]

Identification code	2308601
Empirical formula	$C_{16}H_{23}BiI_2N_2$
Formula weight	706.14
Temperature/K	105.00
Crystal system	monoclinic
Space group	$P2_1/c$
a/Å	8.5281(9)
b/Å	23.554(2)
c/Å	10.0677(10)
α/°	90
β/°	96.084(4)
γ/°	90
Volume/Å^3	2010.9(4)
Z	4
ρ_{calc}g/cm^3	2.332
μ/mm^{-1}	11.835
F(000)	1288.0
Crystal size/mm^3	0.1 × 0.1 × 0.1
Radiation	MoKα (λ = 0.71073)
2Θ range for data collection/°	4.42 to 52.78
Index ranges	-10 ≤ h ≤ 10, -29 ≤ k ≤ 29, -12 ≤ l ≤ 12
Reflections collected	22135
Independent reflections	4129 [R_{int} = 0.0589, R_{sigma} = 0.0475]
Data/restraints/parameters	4129/0/196
Goodness-of-fit on F^2	1.215
Final R indexes [I>=2σ (I)]	R_1 = 0.0663, wR_2 = 0.1683
Final R indexes [all data]	R_1 = 0.1115, wR_2 = 0.2174
Largest diff. peak/hole / e Å^{-3}	7.27/-3.06

[a] Residual electron density maxima (PLAT971) in proximity to I1 and I2 arise due to unsolvable twinning. Residual electron densities maxima (PLAT972) and minima (PLAT973) in proximity to Bi1 are heavy atom specific.

Computational Details

The calculations reported herein were performed using the Turbomole 7.7 program.[10] The crystallographic and fully optimized geometries were used for the calculations of the supramolecular assemblies. We used the crystallographic coordinates as starting points for the gas phase and the periodic boundary condition (PBC) calculations. The level of theory used for the calculations was PBE0[11]-D4[12]/def2-TZVP.[13] For iodine and bismuth, this basis set includes effective core potentials (ECP) and takes into consideration relativistic effects for the inner electrons.[13b] For the model system PhBi, the singlet state instead of triplet was considered. The formation energies of the assemblies have been calculated by subtracting the electronic energy of the assemblies from the total electronic energy of the corresponding monomers. The MEP surface plots were generated using the wavefunction obtained at the same level of theory and the 0.001 a.u. isosurface to simulate the van der Waals envelope. The topological analysis of the electron density was carried out according to the quantum theory of atoms in molecules (QTAIM) method proposed by Bader[14] and the reduced density gradient (RDG) isosurfaces (NCIplot)[15] and represented using the VMD program.[16] The electron localization function (ELF)[17] analysis was performed using the MultiWFN program[18] at the PBE0-D4/def2-TZVP level of theory. The NBO analysis[19] was performed using the same level of theory and the NBO7.0 program.[20]

TD-DFT calculations were performed using Turbomole 7.7 program at the same level of theory for consistency. For the calculations, we evaluated up to five excited states. Since we were interested in the visible region of the spectrum, additional excited states were not calculated for computational economy.

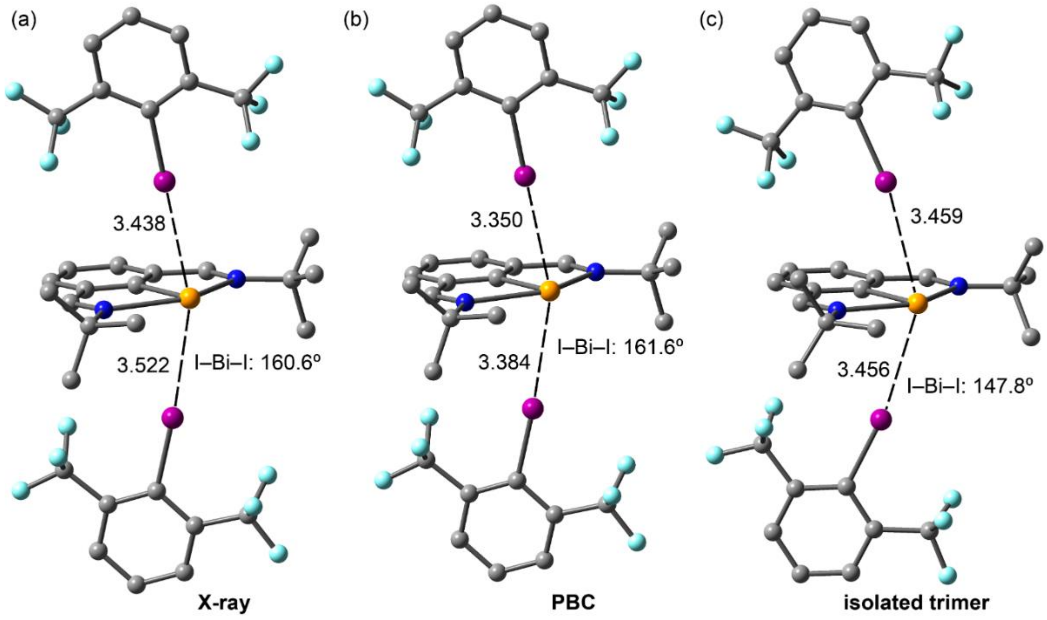

Figure S3: (a) X-ray assembly. (b) PBC optimized trimer. (c) Optimized geometry of the isolated trimer in the gas phase. Distances in Å.

In Figure S4, we have depicted the molecular orbitals of compound PhBi in its singlet state, highlighting the locations of the lone pairs (LPs). It can be observed that one of

the LPs is located in a p orbital (HOMO), while the other LP is in an s orbital (inner lone pair), positioned at HOMO-11.

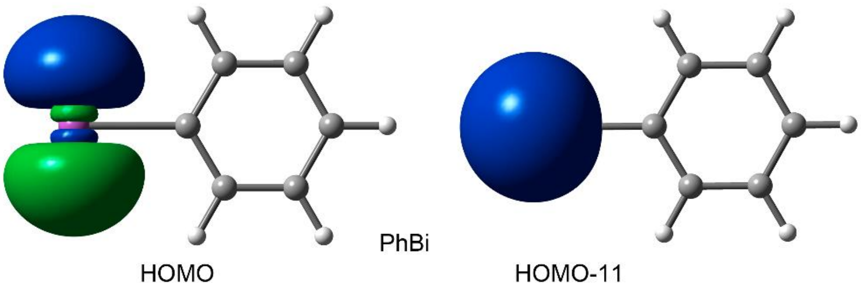

Figure S4. MOs corresponding to the LPs at the Bi atom in PhBi compound

Figure S5 shows the small electron donation from the bismuth-carbon σ bond [σ(Bi–C] to the antibonding σ*(C–I) orbitals. The σ(Bi–C)→σ*(C–I) charge transfer energies are 0.23 and 0.07 kcal/mol.

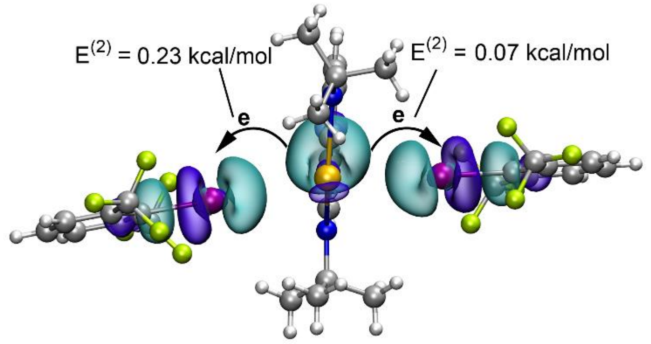

Figure S5. NBOs involved in the σ(Bi–C)→ σ*(C–I) charge transfer in compound **3**. The second order perturbation energies $E^{(2)}$ are indicated.

Cartesian Coordinates

Optimized geometry of the isolated adduct **3**

Bi	9.6772548	7.6462599	9.8742388
I	12.8888312	7.7362259	11.1559894
I	6.5375074	6.2015414	9.9421434
F	14.9865531	10.1918013	10.6985529
F	12.6836949	6.6851607	14.2193110
F	15.5581260	8.5330449	9.4389323
F	3.7977616	7.7331365	9.0249577
F	6.8691720	3.2998787	11.3383099
F	4.3709545	6.3074369	7.5068981
F	6.2764235	4.7347972	12.8404569
F	17.0591383	9.7608819	10.3557765
F	13.2666136	5.0454411	12.9412414
F	2.2990756	6.6377377	7.9519965
F	14.0762593	5.2132449	14.9184490
F	5.5356657	2.7236086	12.9140569
N	10.4327303	6.3946882	7.8474015
N	9.2407851	7.4022732	12.2541629
C	10.0883665	5.6067636	10.4215945
C	10.6953419	5.1721209	8.1169827
H	11.0452855	4.4748135	7.3554790
C	10.5311281	4.6893786	9.4679908
C	10.7975850	3.3760816	9.8448701
H	11.1428886	2.6607474	9.1043869
C	4.7080682	5.1401605	10.2767479
C	10.5761547	6.9667154	6.5083396
C	3.5508005	5.4387696	9.5519612
C	15.8873684	8.2807120	11.7688973
C	9.9160212	5.2063906	11.7474100
C	14.7581908	7.5716972	12.1886789

C	9.4621679	6.2095410	12.6720249
H	9.3129660	5.9330229	13.7166316
C	8.5838230	9.7256712	12.3859485
H	9.5278990	10.0423495	11.9352342
H	8.2345227	10.5296278	13.0363334
H	7.8431842	9.5850560	11.5944332
C	2.3761162	4.7312011	9.7734360
H	1.4932006	4.9784219	9.2003010
C	8.7700778	8.4428913	13.1836660
C	4.6471892	4.1195843	11.2312564
C	3.4649284	3.4208514	11.4415167
H	3.4395122	2.6338924	12.1826610
C	15.8627359	9.1917508	10.5628892
C	2.3306032	3.7218549	10.7143672
H	1.4131291	3.1722012	10.8819881
C	17.0901603	8.1658281	12.4543496
H	17.9504032	8.7237567	12.1110517
C	10.6238265	2.9783741	11.1652579
H	10.8324877	1.9556217	11.4532203
C	3.5143746	6.5326933	8.5087619
C	14.8755189	6.7450063	13.3109016
C	7.4322311	8.0239953	13.7883376
H	6.7042157	7.8227731	13.0002425
H	7.0456639	8.8210711	14.4272260
H	7.5308537	7.1230176	14.3967503
C	11.5545138	8.1343861	6.6266239
H	11.1944484	8.8725748	7.3459129
H	11.6732896	8.6274129	5.6592235
H	12.5329781	7.7872646	6.9647221
C	17.1918769	7.3489854	13.5627498
H	18.1315134	7.2630842	14.0935102
C	10.1848272	3.8887340	12.1139491
H	10.0512133	3.5775116	13.1450727
C	9.1980857	7.4798985	6.0934253
H	8.4794846	6.6593946	6.0404020
H	9.2524891	7.9603903	5.1141529
H	8.8212702	8.2088190	6.8136054
C	13.7190704	5.9247679	13.8385707
C	16.0850508	6.6408931	13.9867438
H	16.1545743	5.9960186	14.8518491
C	5.8398951	3.7248656	12.0743830
C	9.8176432	8.6679955	14.2708769
H	9.9555055	7.7760223	14.8852276
H	9.5071980	9.4844729	14.9264187
H	10.7807573	8.9237479	13.8251305
C	11.0868623	5.9789006	5.4676358
H	12.0769252	5.5948933	5.7252971
H	11.1690849	6.4853274	4.5046000
H	10.4048954	5.1348673	5.3397051

PBC optimized adduct **3**

F	5.1867856	4.9117973	12.8821457
C	4.5716482	4.0293888	12.0462930
F	3.8639173	3.1818617	12.8534799
F	5.5519269	3.2684500	11.4760365
C	3.6862776	4.7029434	11.0175674
C	2.3232406	4.3733044	11.0237648
H	1.9466696	3.6714061	11.7629122
C	1.4531856	4.9326176	10.0959919
H	0.3931743	4.6846266	10.1232760
C	1.9470434	5.8101933	9.1382910
H	1.2769804	6.2509894	8.4034296
C	4.1942917	5.6070879	10.0673849
C	3.3090855	6.1425130	9.1137127
C	3.7788569	7.0696406	8.0131201
F	2.7785211	7.4161632	7.1675954
F	4.2979644	8.2452128	8.4952680
F	4.7544326	6.5013976	7.2419494
I	6.3551190	6.1860310	10.0636413
Bi	9.6129385	7.1040407	10.1231907
C	10.3431076	5.1164366	10.6814046
C	10.2464816	4.6983863	12.0207459
C	10.7849738	3.4528740	12.3942069
H	10.7140955	3.1244247	13.4313926
C	11.3996413	2.6378606	11.4392169
H	11.8221502	1.6748592	11.7281617
C	11.4903155	3.0523097	10.1046901
H	11.9843392	2.4153755	9.3697528
C	10.9696320	4.3033095	9.7198666
C	9.6304689	5.6192846	12.9541169
H	9.5454773	5.3316190	14.0097869
N	9.2048378	6.7699894	12.5295332
C	11.0791581	4.8415838	8.3738633
H	11.5752200	4.2425444	7.5998499
N	10.6068780	6.0234427	8.1235621
C	8.6341901	7.7666352	13.4773710

C	7.5080798	7.1419423	14.3145704
H	7.8870499	6.3628972	14.9890152
H	7.0347640	7.9152065	14.9370385
H	6.7500713	6.6883412	13.6637134
C	8.0848759	8.9300610	12.6437798
H	7.3143449	8.5779243	11.9424118
C	9.7735955	8.2567589	14.3789752
H	10.5875303	8.6655517	13.7676844
H	9.4200166	9.0316947	15.0690762
H	10.1766969	7.4259148	14.9724621
C	10.7668583	6.6662455	6.7912503
C	10.4747282	5.6834452	5.6459595
H	10.5508853	6.2107446	4.6857926
H	9.4544924	5.2831222	5.7347686
H	11.1784855	4.8405208	5.6187585
C	12.2060320	7.2034922	6.7018179
H	12.3329922	7.7831350	5.7786051
C	9.7669707	7.8256575	6.7304819
H	10.0020514	8.5863913	7.4901443
H	9.8018984	8.3213623	5.7517436
H	8.7445440	7.4597550	6.8985194
H	7.6407931	9.6935072	13.2951902
H	12.9297610	6.3767744	6.6963985
H	12.4250475	7.8595727	7.5557290
H	8.8899242	9.4132437	12.0694336
F	0.3695779	5.3348845	13.1304088
C	0.8788985	6.3632761	13.8683420
F	-0.1931943	7.0442596	14.3718683
F	1.4983327	5.7903802	14.9371104
C	1.8148236	7.2716446	13.0946909
C	3.1483543	7.3381214	13.5271271
H	3.4757015	6.7247901	14.3640471
C	4.0544482	8.1790830	12.8892588
H	5.0909549	8.2151394	13.2187567
C	3.6293238	8.9702183	11.8275621
C	2.2978522	8.9150198	11.3912306
C	1.3740650	8.0526770	12.0097549
H	4.3287971	9.6390132	11.3300227
C	1.8944284	9.8413039	10.2601181
F	1.4646882	9.1767145	9.1544571
F	0.8897173	10.6879984	10.6359860
F	2.9290180	10.6310497	9.8606080
I	-0.7221963	7.8323231	11.2238277
H	-1.7376703	6.0952981	15.8300845
C	-1.8085558	5.7668359	16.8672768
C	-2.4232332	6.5818485	17.8222608
H	-2.8457170	7.5448500	17.5333158
C	-2.5138961	6.1673775	19.1567475
H	-3.0079370	6.8043327	19.8917231
C	-1.2701033	4.5213079	17.2406999
C	-1.9932425	4.9164038	19.5415898
C	-1.3666913	4.1032348	18.5800599
C	-0.6540470	3.6004072	16.3073601
H	-0.5690735	3.8880988	15.2516754
N	-0.2284486	2.4497217	16.7319330
C	-2.1027349	4.3780983	20.8876043
H	-2.5988076	4.9771501	21.6616009
N	-1.6304587	3.1962440	21.1378910
C	0.3422198	1.4530696	15.7840851
C	1.4683194	2.0777725	14.9468722
H	1.0893369	2.8568128	14.2724150
H	1.9416354	1.3045007	14.3244052
H	2.2263428	2.5313740	15.5977227
C	0.8915425	0.2896478	16.6176734
H	1.6620825	0.6417957	17.3190430
C	-0.7971980	0.9629488	14.8824820
H	-1.6111289	0.5541531	15.4937842
H	-0.4436109	0.1880130	14.1923719
H	-1.2003043	1.7938065	14.2890014
C	-1.7904543	2.5534345	22.4702060
C	-1.4983139	3.5362291	23.6154902
H	-1.5744649	3.0089399	24.5756657
H	-0.4780677	3.9365488	23.5266804
H	-2.2020683	4.3791623	23.6426950
C	-3.2296381	2.0162041	22.5596373
H	-3.3565781	1.4365588	23.4828497
C	-0.7905709	1.3940287	22.5309636
H	-1.0256656	0.6332879	21.7713025
H	-0.8254934	0.8983224	23.5097046
H	0.2318686	1.7599220	22.3629225
H	1.3356302	-0.4737945	15.9662529
H	-3.9533631	2.8429250	22.5650636
H	-3.4486482	1.3601197	21.7057287
H	0.0864823	-0.1935348	17.1920219
Bi	-0.6365388	2.1156458	19.1382654
I	2.6212821	3.0336610	19.1978200
C	4.7821154	3.6126087	19.1940658
C	5.2901267	4.5167377	18.2439126

C	6.6531534	4.8464063	18.2376682
H	7.0297241	5.5482932	17.4985453
C	7.5232313	4.2870785	19.1654770
H	8.5832190	4.5350798	19.1381669
C	7.0293542	3.4094969	20.1231615
H	7.6994379	2.9687231	20.8580140
C	5.6673221	3.0771963	20.1477295
C	5.1975647	2.1500636	21.2483116
F	6.1978922	1.8035294	22.0938517
F	4.6784617	0.9744879	20.7661801
F	4.2220115	2.7182925	22.0195034
C	4.4047598	5.1902920	17.2151788
F	3.7896257	4.3078942	16.3793070
F	5.1124709	6.0378332	16.4079886
F	3.4244878	5.9512504	17.7854208
F	8.6068488	3.8848251	16.1310496
C	8.0974893	2.8564399	15.3931228
F	9.1695685	2.1754593	14.8895900
F	7.4780779	3.4293227	14.3243369
C	7.1616075	1.9480266	16.1667676
C	5.8280299	1.8816130	15.7343186
H	5.5007024	2.4949281	14.8974049
C	4.9219643	1.0406186	16.3721989
H	3.8854406	1.0045773	16.0426853
C	5.3470625	0.2495005	17.4338876
C	6.6785829	0.3046870	17.8701822
C	7.6023576	1.1670460	17.2517046
H	4.6475984	-0.4193077	17.9314120
C	7.0819623	-0.6215908	19.0013135
F	7.5117325	0.0429636	20.1069757
F	8.0866620	-1.4682782	18.6254428
F	6.0473939	-1.4113674	19.4008233
I	9.6986224	1.3873743	18.0376288
F	7.4172217	3.1984899	24.4912652
C	6.3826514	3.9882190	24.8908110
F	5.9529036	4.6527848	23.7851843
F	5.3778726	3.1415191	25.2667046
C	6.7860500	4.9145611	26.0217622
C	8.1175143	4.8593033	26.4583147
H	8.8170447	4.1904915	25.9607325
C	8.5427436	5.6505086	27.5199806
C	7.6365081	6.4913594	28.1579493
H	7.9638892	7.1048489	28.9949274
C	6.3029572	6.5581598	27.7252001
C	5.8622031	5.7767525	26.6404889
C	5.3671178	7.4662745	28.4991366
F	4.2948744	6.7853075	29.0026174
F	4.8577819	8.4946811	27.7612293
H	9.5793543	5.6144856	27.8494786
F	5.9865257	8.0393368	29.5678736
I	3.7659847	5.9971791	25.8546500
Bi	0.6872881	6.7255124	24.7541305
C	1.4174552	8.7130911	25.3120955
C	1.3210953	9.1310599	26.6514760
C	0.7046743	8.2102806	27.5848916
H	0.6198712	8.4979535	28.6407286
N	0.2792340	7.0594194	27.1602037
C	-0.2915160	6.0628640	28.1080281
C	-1.4173595	6.6875839	28.9452756
H	-2.1755512	7.1412680	28.2944406
C	-0.8407061	4.8995529	27.2745746
H	-1.6113226	5.2515810	26.5731009
H	-1.2848819	4.1360077	27.9260359
H	-0.0356413	4.4162546	26.7001263
C	0.8478477	5.5728332	29.0096283
H	1.6619683	5.1639928	28.3983852
H	-1.0383997	7.4667379	29.6198108
H	0.4943394	4.7977974	29.6998546
H	1.2510919	6.4037251	29.6031510
H	-1.8908472	5.9143106	29.5678052
C	1.8593875	10.3768177	27.0249297
H	1.7885871	10.7051875	28.0622770
C	2.4739995	11.1918064	26.0698411
H	2.8966085	12.1549493	26.3588245
C	2.5646534	10.7771766	24.7353411
H	3.0587045	11.4141645	24.0004303
C	2.0440830	9.5261090	24.3504100
C	2.1535158	8.9878250	23.0044390
N	1.6812793	7.8060707	22.7541748
H	2.6496098	9.5869963	22.2305754
C	1.8413084	7.1632680	21.4219703
C	1.5490742	8.1460523	20.2766610
H	1.6252425	7.6188440	19.3165904
H	0.5288908	8.5463316	20.3654658
H	2.2527627	8.9889294	20.2494445
C	3.2804433	6.6260527	21.3325498
H	3.4074027	6.0464707	20.4094589
H	4.0041222	7.4526733	21.3271240

H	3.4994872	5.9700186	22.1863921
C	0.8414409	6.0038804	21.3612189
H	1.0764385	5.2431755	22.1208697
H	0.8762962	5.5081574	20.3826055
H	-0.1809577	6.3696640	21.5292995
I	-2.5704604	7.6435044	24.6943983
C	-4.7313561	8.2223232	24.6980690
C	-5.2392401	9.1266526	25.6482147
C	-6.6023901	9.4563001	25.6544850
H	-6.9789079	10.1582783	26.3937317
C	-7.4724885	8.8969072	24.7267233
H	-8.5324997	9.1449587	24.7540653
C	-6.9785883	8.0193272	23.7689963
H	-7.6486276	7.5786072	23.0341967
C	-5.6165401	7.6869437	23.7442864
C	-5.1467798	6.7598722	22.6437842
F	-6.1471094	6.4133593	21.7983035
F	-4.6276346	5.5843091	23.1260444
F	-4.1711845	7.3281611	21.8726655
C	-4.3539842	9.8001915	26.6770214
F	-3.7387713	8.9177378	27.5129048
F	-5.0616529	10.6477756	27.4842711
F	-3.3736390	10.5611174	26.1067763
F	1.5591805	6.0212252	4.7702125
C	2.5937491	5.2314847	4.3706885
F	3.0235225	4.5668955	5.4763072
F	3.5985287	6.0781789	3.9947799
C	2.1903580	4.3051570	3.2397137
C	0.8588856	4.3604107	2.8031549
H	0.1593626	5.0292201	3.3007484
C	0.4336491	3.5691897	1.7414756
C	1.3399027	2.7283489	1.1034982
H	1.0125135	2.1148717	0.2665210
C	2.6734625	2.6615334	1.5362401
C	3.1142016	3.4429533	2.6209713
C	3.6092930	1.7534123	0.7623123
F	4.6815224	2.4343979	0.2588471
F	4.1186394	0.7250100	1.5002380
H	-0.6029521	3.6052419	1.4119796
F	2.9898807	1.1803831	-0.3064422
I	5.2104240	3.2225227	3.4068086
Bi	8.2891049	2.4941960	4.5073308
C	7.5589507	0.5065991	3.9493351
C	7.6553295	0.0886214	2.6099649
C	8.2717324	1.0094528	1.6765659
H	8.3565273	0.7217632	0.6207286
N	8.6971907	2.1602684	2.1012401
C	9.2679106	3.1568280	1.1534403
C	10.3937794	2.5321176	0.3161870
H	11.1519592	2.0784447	0.9670332
C	9.8171044	4.3201589	1.9868986
H	10.5877186	3.9681254	2.6883643
H	10.2612714	5.0837013	1.3354282
H	9.0120347	4.8034498	2.5613388
C	8.1285477	3.6468720	0.2518174
H	7.3144384	4.0557100	0.8630702
H	10.0148122	1.7529626	-0.3583461
H	8.4820758	4.4219179	-0.4384033
H	7.7253290	2.8159694	-0.3417101
H	10.8672568	3.3054035	-0.3063403
C	7.1170475	-1.1570790	2.2364901
H	7.1877872	-1.4854731	1.1991566
C	6.5023520	-1.9721348	3.1915903
H	6.0797692	-2.9352382	2.9026117
C	6.4117824	-1.5574673	4.5261244
H	5.9176828	-2.1944736	5.2610267
C	6.9323148	-0.3064343	4.9110397
C	6.8228897	0.2318945	6.2570164
N	7.2951129	1.4136201	6.5072866
H	6.3267805	-0.3673027	7.0308700
C	7.1351009	2.0564221	7.8394723
C	7.4273182	1.0736251	8.9847858
H	7.3511499	1.6008443	9.9448461
H	8.4474963	0.6733435	8.8960048
H	6.7236244	0.2307491	9.0120160
C	5.6959687	2.5936446	7.9289103
H	5.5689963	3.1732329	8.8520072
H	4.9722899	1.7670181	7.9343267
H	5.4769336	3.2496736	7.0750570
C	8.1349779	3.2158157	7.9002426
H	7.8999747	3.9765104	7.1405865
H	8.1001033	3.7115191	8.8788573
H	9.1573620	2.8500158	7.7321679
I	11.5468863	1.5761795	4.5670539
C	13.7077836	0.9973491	4.5633782
C	14.2156675	0.0930108	3.6132402
C	15.5787826	-0.2365951	3.6069694
H	15.9553181	-0.9385669	2.8677329

C	16.4488910	0.3228209	4.5347533
H	17.5088995	0.0747510	4.5073994
C	15.9549652	1.2003480	5.4924195
H	16.6250187	1.6411034	6.2272758
C	14.5929865	1.5327438	5.5172097
C	14.1231506	2.4598401	6.6176700
F	15.1234876	2.8063099	7.4631521
F	13.6040116	3.6354204	6.1354126
F	13.1475821	1.8915336	7.3887979
C	13.3303763	-0.5804859	2.5844124
F	12.7151549	0.3019698	1.7485285
F	14.0380552	-1.4280816	1.7771977
F	12.3500563	-1.3414300	3.1546695

Et_3Bi optimized

Bi	1.4595312	0.9908073	1.3291658
C	0.6445739	2.8631949	0.3318244
C	0.8784998	2.9738025	-1.1603545
H	1.0980022	3.6988327	0.8708155
H	-0.4217674	2.8717968	0.5713173
H	0.4621744	3.9045206	-1.5615194
H	1.9428010	2.9663890	-1.4068025
H	0.4110016	2.1516627	-1.7067778
C	0.5413332	-0.4515388	-0.1567523
C	1.0712384	-1.8618159	0.0213869
H	0.7623313	-0.0653527	-1.1534397
H	-0.5407442	-0.4082752	-0.0130669
H	0.6192201	-2.5569359	-0.6941909
H	2.1535804	-1.9074917	-0.1274471
H	0.8642138	-2.2502274	1.0224697
C	3.4404369	1.0576981	0.2308426
C	4.2730043	2.2668891	0.6105481
H	3.2120407	1.0395759	-0.8369951
H	3.9568120	0.1262880	0.4743095
H	5.2341512	2.2785876	0.0857828
H	3.7616896	3.2023096	0.3666256
H	4.4909644	2.2876580	1.6821424

Ph_3Bi optimized

Bi	-0.4824425	0.8004523	1.7069024
C	1.4778564	0.8023698	0.6155745
C	1.6359277	0.8047831	-0.7656894
C	2.6159346	0.8010138	1.4163323
C	2.9013717	0.8063017	-1.3337251
H	0.7753186	0.8054534	-1.4226787
C	3.8852177	0.8023330	0.8526744
H	2.5226894	0.7988991	2.4989667
C	4.0288862	0.8051999	-0.5254161
H	3.0069501	0.8083559	-2.4131007
H	4.7609420	0.8011239	1.4920303
H	5.0173952	0.8065360	-0.9702857
C	-1.4616978	-0.8942895	0.6085631
C	-2.0303972	-1.8808252	1.4085311
C	-1.5446316	-1.0287394	-0.7729642
C	-2.6698966	-2.9769137	0.8444317
H	-1.9797134	-1.8032405	2.4911855
C	-2.1820788	-2.1218606	-1.3414874
H	-1.1142093	-0.2837054	-1.4301973
C	-2.7469995	-3.0981076	-0.5336237
H	-3.1080792	-3.7353269	1.4837945
H	-2.2382581	-2.2110044	-2.4208406
H	-3.2462402	-3.9511794	-0.9786418
C	-1.4621586	2.4996763	0.6157797
C	-1.5434606	2.6404239	-0.7651673
C	-2.0325876	3.4820924	1.4195957
C	-2.1810189	3.7356548	-1.3295004
H	-1.1114551	1.8988259	-1.4252730
C	-2.6717247	4.5806817	0.8597010
H	-1.9836398	3.3994454	2.5019225
C	-2.7471578	4.7080513	-0.5179019
H	-2.2362140	3.8294739	-2.4084976
H	-3.1107385	5.3361068	1.5019048
H	-3.2460159	5.5630595	-0.9596533

PhBi optimized

Bi	-2.5511417	1.3699191	0.0180843
C	-0.3450931	1.3700447	0.0175471
C	0.3714822	0.1670479	0.0598256
C	0.3712172	2.5732222	-0.0245596
C	1.7571399	0.1669939	0.0612482
H	-0.1691425	-0.7721566	0.0923599
C	1.7568835	2.5736099	-0.0253545
H	-0.1696386	3.5123145	-0.0573643
C	2.4486437	1.3703836	0.0181643

H	2.3004988	-0.7706221	0.0956463
H	2.3000338	3.5113856	-0.0595213
H	3.5330028	1.3705251	0.0185228

1a optimized

Bi	9.6690883	7.0998758	10.1386490
N	10.6571257	6.0582571	8.1255577
N	9.2257922	6.7420886	12.5262089
C	10.3623445	5.1560971	10.6589278
C	11.0947499	4.8719017	8.3694944
H	11.5746706	4.2638355	7.6011159
C	10.9611292	4.3315312	9.6911351
C	11.4026707	3.0585904	10.0420717
H	11.8660261	2.4241188	9.2919285
C	10.8410575	6.6694984	6.8029446
C	10.2206336	4.6855686	11.9758248
C	9.6179159	5.5782723	12.9196056
H	9.5110191	5.2553834	13.9564739
C	8.0944818	8.8733869	12.7035398
H	8.8851342	9.3612942	12.1283335
H	7.6644059	9.6125326	13.3823488
H	7.3146606	8.5459374	12.0121299
C	8.6512365	7.6932961	13.4881835
C	11.2566996	2.5975125	11.3455824
H	11.6043982	1.6068687	11.6114884
C	7.5220568	7.0507772	14.2906360
H	6.7578722	6.6519933	13.6205285
H	7.0575981	7.7948839	14.9413496
H	7.8812745	6.2383867	14.9250131
C	12.2805092	7.1732987	6.7057201
H	12.4964257	7.8648288	7.5220974
H	12.4410032	7.6906151	5.7567150
H	12.9860357	6.3415066	6.7685792
C	10.6693207	3.4089788	12.3079982
H	10.5598590	3.0485014	13.3269336
C	9.8809855	7.8478771	6.7013022
H	8.8459439	7.5126118	6.7952246
H	9.9964467	8.3529091	5.7399884
H	10.0811016	8.5765295	7.4907177
C	9.7584733	8.1811346	14.4206538
H	10.1654419	7.3561286	15.0089847
H	9.3722938	8.9354521	15.1103654
H	10.5731072	8.6220700	13.8427850
C	10.5459793	5.6899124	5.6690340
H	11.2700720	4.8742111	5.6250843
H	10.5894813	6.2134329	4.7117157
H	9.5478783	5.2613130	5.7820293ç

1b optimized

Bi	9.8172370	7.1327093	10.1789320
N	10.7424740	6.0765098	8.1782660
N	9.3171637	6.7400155	12.5390811
C	10.4012095	5.1407884	10.6731204
C	11.1247781	4.8610080	8.3869416
H	11.5586362	4.2672766	7.5803587
C	10.9698585	4.3036663	9.6961157
C	11.3484045	3.0048863	10.0279784
H	11.7875892	2.3620038	9.2708031
C	10.2219560	4.6504847	11.9793034
C	9.6378632	5.5507672	12.9265182
H	9.4664335	5.2358347	13.9575056
C	11.1676457	2.5285851	11.3207741
H	11.4642982	1.5177870	11.5716819
C	10.6064974	3.3487989	12.2920185
H	10.4667666	2.9742001	13.3018280
C	8.7309351	7.6536290	13.4421437
C	7.3398730	7.6971914	13.5657251
C	9.5479816	8.5426696	14.1444938
C	6.7836832	8.6402993	14.4195556
C	8.9439342	9.4672932	14.9865066
C	7.5657075	9.5319659	15.1422388
H	5.7026784	8.6806733	14.5164186
H	9.5742052	10.1625517	15.5332732
C	10.8773790	6.6594895	6.8991285
C	9.8215626	6.5657962	5.9891986
C	12.0361298	7.3764513	6.5913469
C	9.9553758	7.1931634	4.7576057
C	12.1223457	7.9858893	5.3464792
C	11.0969646	7.9049016	4.4135380
H	9.1359011	7.1260311	4.0479910
H	13.0198924	8.5466949	5.1025758
C	8.5789661	5.8083101	6.3379796
H	8.7717196	4.7343243	6.4145245
H	8.1824077	6.1222524	7.3068539
H	7.8102314	5.9580814	5.5794361

C	13.1526573	7.4843632	7.5822139
H	12.7900246	7.8395584	8.5502992
H	13.6233080	6.5141005	7.7645173
H	13.9214771	8.1692911	7.2239139
C	11.2274325	8.5485131	3.0665860
H	11.6706494	7.8575033	2.3425604
H	10.2546221	8.8510271	2.6746047
H	11.8672903	9.4319674	3.1076901
C	6.4751953	6.7503950	12.7931939
H	6.7490581	6.7373413	11.7351700
H	6.5813830	5.7236056	13.1550476
H	5.4247445	7.0293602	12.8799588
C	11.0359911	8.4960899	13.9906094
H	11.4529966	7.5724306	14.4019349
H	11.3270096	8.5256290	12.9374314
H	11.5037984	9.3351402	14.5063338
C	6.9404859	10.5207447	16.0784132
H	5.9628016	10.8462179	15.7182565
H	6.7930910	10.0829858	17.0707793
H	7.5693834	11.4041410	16.2017346

References

[1] a) I. Vránová, M. Alonso, R. Lo, R. Sedlák, R. Jambor, A. Růžička, F. de Proft, P. Hobza, L. Dostál, *Chem. Eur. J.* **2015**, *21*, 16917-16928; b) T. Tsuruta, D. Spinnato, H. W. Moon, M. Leutzsch, J. Cornella, *J. Am. Chem. Soc.*, **2023**, *145*, 25538-25544.

[2] F. Rauch, S. Fuchs, A. Friedrich, D. Sieh, I. Krummenacher, H. Braunschweig, M. Finze, T. B. Marder, *Chem. Eur. J.* **2020**, *26*, 12794-12808.

[3] a) M. Hejda, R. Jirásko, A. Růžička, R. Jambor, L. Dostál, *Organometallics* **2020**, *39*, 4320-4328; b) M. Mato, P. C. Bruzzese, F. Takahashi, M. Leutzsch, E. J. Reijerse, A. Schnegg, J. Cornella, *J. Am. Chem. Soc.* **2023**, *145*, 18742-18747.

[4] O. V. Dolomanov, L. J. Bourhis, R. J. Gildea, J. A. K. Howard, H. Puschmann, *J. Appl. Cryst.* **2009**, *42*, 339-341.

[5] G. M. Sheldrick, *Acta Cryst.* **2015**, *C71*, 3-8.

[6] a) G. M. Sheldrick, **2014**; b) G. Sheldrick, *Acta Cryst.* **2008**, *A64*, 112-122.

[7] Bonn, Germany.

[8] C. F. Macrae, P. R. Edgington, P. McCabe, E. Pidcock, G. P. Shields, R. Taylor, M. Towler, J. van de Streek, *J. Appl. Cryst.* **2006**, *39*, 453-457.

[9] Persistence of Vision Pty. Ltd. Persistence of Vision Raytracer. Ltd., Persistence of Vision Pty. 2004. Retrieved from http://www.povray.org/download/.

[10] R. Ahlrichs, M. Bär, M. Häser, H. Horn, C. Kölmel, *Chem. Phys. Lett.* **1989**, *162*, 165-169.

[11] C. Adamo, V. Barone, *J. Chem. Phys.* **1999**, *110*, 6158-6170.

[12] S. Grimme, J. Antony, S. Ehrlich, H. Krieg, *J. Chem. Phys.* **2010**, *132*, 154104.

[13] a) F. Weigend, *Phys. Chem. Chem. Phys* **2006**, *8*, 1057-1065 ; b) F. Weigend, R. Ahlrichs, *Phys. Chem. Chem. Phys* **2005**, *7*, 3297-3305

[14] R. F. W. Bader, *Chem. Rev.* **1991**, *91*, 893-928.

[15] J. Contreras-García, E. R. Johnson, S. Keinan, R. Chaudret, J.-P.-. Piquiemal, D. N. Beratan, W. Yang, *J. Chem. Theory Comput.* **2011**, *7*, 625-632.

[16] W. Humphrey, A. Dalke, K. Schulten, *J. Molec. Graphics* **1996**, *14*, 33-38.

[17] A. D. Becke, K. E. Edgecombe, *J. Chem. Phys.* **1990**, *92*, 5397-5403.

[18] T. Lu, F. Chen, *J. Comput. Chem.* **2012**, *33*, 580-592.

[19] E. D. Glendening, C. R. Landis, F. Weinhold, *J. Comput. Chem.* **2019**, *40*, 2234-2241.

[20] Glendening, E. D. B., J. K.; Reed, A. E.; Carpenter, J. E.; Bohmann, J. A.; Morales, C. M.; Karafiloglou, P.; Landis, C. R.; Weinhold F. NBO 7.0., Theoretical Chemistry Institute, University of Wisconsin, Madison, WI., 2018.
